# Association of lifestyle and disease characteristics with self-rated wellness/health score in patients with rheumatoid arthritis

**DOI:** 10.1186/s41927-021-00227-x

**Published:** 2021-12-22

**Authors:** Reihane Tabaraii, Maryam Masoumi, Mahsa Bagherzadeh-Fard, Mohammad Amin Yazdanifar, Javad Balasi, Abbas Smiley

**Affiliations:** 1 Clinical Research Development Center, Qom University of Medical Sciences, Qom, Iran; 2 School of Medicine, Iran University of Medical Sciences, Tehran, Iran; 3 Westchester Medical Center, New York Medical College, 100 Woods, Valhalla, NY 10595 USA

**Keywords:** Rheumatoid arthritis, Sleep, Mood, Self-rated wellness/health score

## Abstract

**Objective:**

To study the relationship of self-rated wellness/health and lifestyle in patients with rheumatoid arthritis.

**Methods:**

Self-rated wellness/health, demographics, smoking, mood, sleep, physical activity, diet, symptoms/signs, body mass index and laboratory findings in 142 patients with rheumatoid arthritis were collected in the current cross-sectional study. Multivariable generalized additive model (GAM) was employed to study the association of self-rated wellness/health score and lifestyle factors.

**Results:**

Female/male ratio was 116/26 and the mean (SD) age of sample was 52 (13) years. Mean (SD) self-rated wellness/health score out of 10 was 7.2 (1.63). Mean (SD) number of tender joints and swollen joints were 4.42 (4.55) and 4.00 (4.26), respectively. The mean sleep score was 29.5 out of 70. Patients went to bed more than one hour earlier during the weekdays compared to weekends (22:45 vs. 23:52 PM, respectively, *p* < 0.0001). They also woke up more than one hour earlier during the weekdays compared to the weekends (6:08 vs. 7:20 AM, respectively, *p* < 0.0001). Their nap duration during weekdays was about half an hour shorter than the nap duration on weekends (19.75 vs. 48.02 minutes, respectively, *p* < 0.0001). The mean mood and diet scores were 18.5/35 and 22.5/42, respectively. By backward elimination in multivariable regression model (GAM), disease duration, mood, sleep quality, weekdays sleep characteristics (sleep duration, time to go to bed, wake-up time, time to fall asleep and nap duration), and sleep duration on weekends remained in the final model (R^2^ = 0.225, *p* = 0.01). Sleep quality, nap duration on weekdays, night sleep duration on weekends and mood status were the significant variables associated with self-rated wellness/health score.

**Conclusion:**

In patients with rheumatoid arthritis, the low self-rated wellness/health score was associated with the low sleep quality, long sleep duration on weekends, and long nap duration on weekdays.

## Introduction

Several lifestyle factors have been associated with increased/decreased inflammation or the severity of inflammatory disorders. The most important ones are physical activity [1], fast foods [2], omega 3 [3], smoking [4], sleep [5], social activity [6], and stress [7, 8]. Rheumatoid arthritis is a chronic systemic inflammation mainly targeting the joints [9]. Investigations have shown the association of rheumatoid arthritis with comorbidities and several dimensions of lifestyle such as poor sleep [10, 11], high prevalence of depression/anxiety in patients with rheumatoid arthritis [12]. Studies have shown negative impacts of smoking [13] and fast foods [14] on rheumatoid arthritis disease activity. Specifically, important among the lifestyle factors has been sleep insufficiency which has been shown to be associated with the severity of symptoms and signs in patients with rheumatoid arthritis [10, 11].

Self-rated wellness/health has been shown to provide a reliable subjective picture of well-being and health status [15]. In addition to mood status and various lifestyle factors such as sleep [16], diet [17, 18], physical activity [19, 20], social activity [21–23] and smoking [24–26], any chronic painful/limiting disorder such as rheumatoid arthritis can negatively affect the self-rated wellness/health status [27–29]. It seems there are bidirectional relationships among the three sides of triangle: chronic disease manifestations, lifestyle factors and self-rated wellness/health. Then, understanding the complex inter-relationships in this triangle in rheumatoid arthritis might be an important aspect for any patient-centered clinical practice and management plan. Establishing the strengths of associations of this triangle for rheumatoid arthritis needs several investigations. The goal of the current study was to evaluate only one side of the triangle; i.e., the association of self-rated wellness/health score and lifestyle factors in patients with rheumatoid arthritis.

## Methods

142 patients with rheumatoid arthritis were consecutively enrolled in our cross-sectional study which was carried out in a medical university affiliated clinic during the summertime. The convenience sampling method was employed to enroll all the patients conveniently visited the clinic. Information about demographics, smoking, social activity, mood status, sleep quality, sleep duration, physical activity, diet, disease duration and symptoms were inquired and recorded. Body mass index, signs, and laboratory data were actively assessed and recorded. Sleep quality was evaluated by Mini-Sleep Questionnaire [30]. The questionnaire included 10 questions (Table 1) totaling 70 scores altogether. The higher the score, the worst the sleep quality. Extra questions were devoted to measure sleep quantity as well (Table 1). Mood (Table 1) was evaluated according to Gallup Well-being Index [31]. It has 5 questions that are presented in Table 1. The total score was 35 and the higher the score, the worst the mood status. Diet status (Table 1) was assessed using modified Gallup Diet Questionnaire with 6 questions (Table 1). The total score was 42 and again the higher score denoted the worst diet pattern [32–36]. Physical activity (Table 1] was inquired based on a modified question from Brunel lifestyle physical activity questionnaire [37]. Smoking pattern which has strong association with wellbeing and health status was assessed [38]. Self-rated wellness/health score was defined as the last question of questionnaire where 10 being the healthiest state and 0 the unhealthiest (Table 1) [15, 39, 40]. The study protocol was approved by the Institutional Review Board at Clinical Research Development Center affiliated with the Qom University of Medical Sciences. All patients provided informed consent before enrollment. All methods were carried out in accordance with relevant guidelines and regulations in the methods section.

**Table 1:** Lifestyle questionnaire and the calculating method of corresponding scores

Lifestyle factors	Question	Time or days per week
Quantity of sleep	What time did you usually go to bed on weekdays?	
	How long did it take to fall asleep?	
	What time did you usually go to bed on weekends?	
	What time did you usually get out of bed on weekdays?	
	What time did you usually get out of bed on weekends?	
	How many hours did you sleep every night on weekdays?	
	How many hours did you sleep every night on weekends?	
	How many hours did you get a nap on weekdays?	
	How many hours did you get a nap on weekends?	
Sleep quality	How many days per week do you have difficulties falling asleep?	/7
	How many days per week do you wake up too early?	/7
	How many days per week do you use Hypnotic medications (sleep aids)?	/7
	How many days per week do you fall asleep during the day?	/7
	How many days per week do you feel tired upon waking up in the morning?	/7
	How many days per week do you snore?	/7
	How many days per week do you experience mid-sleep awakenings?	/7
	How many days per week do you experience headaches on awakening?	/7
	How many days per week do you experience excessive daytime sleepiness?	/7
	How many days per week do you experience excessive movement during sleep?	/7
Total score of sleep quality out of 70		/70
Mood	How many days per week do you experience no energy to get things done?	/7
	How many days per week do you experience sadness?	/7
	How many days per week do you experience worry?	/7
	How many days per week do you experience anger?	/7
	How many days per week do you experience physical pain?	/7
Total score of mood status out of 35		/35
Diet	How many days per week do you eat fast food?	/7
	How many days per week did you eat red meat?	/7
	How many days per week do you eat fish/omega 3?	/7
	How many days per week do you eat 4–5 servings of fruits/vegetables?	/7
	How many days per week did you take vitamin D tablet?	/7
	How many days per week did you take Magnesium tablet?	/7
Total Score of Diet out of 42		/42
Physical activity	How many days per week in a normal week do you engage in at least 30-minute pre-planned physical activity?	/7
Social activity	How many days per week did you participate in a social, cultural, or support group that you belong to?	/7
Smoking behavior	Do you smoke?	
	If yes, how many cigarettes do you smoke per day?	
Self-rated wellness & health	How much do you rate your wellness and health out of 10; 10 being the healthiest and 0 being the unhealthiest?	/10

### Statistical analysis

The frequency distributions of demographic information, lifestyle, symptoms/signs and lab data were determined using descriptive analyses. Continuous variables were compared using t-test. Continuous variables between weekdays and weekends were compared by paired t-test. Univariable association of self-rated wellness/health score and other assessments was carried out through linear and nonlinear (generalized additive model, GAM) regression analyses. First, all continuous variables were evaluated by GAM to understand which ones had non-linear relationship with self-rated wellness/health score. Sleep quality score, time to go to bed on weekdays, wake-up time on weekdays, nap duration on weekdays, and time to go to bed on weekends showed some degrees of nonlinearity. GAM was used to present their univariable associations. All other variables were evaluated univariably by generalized linear model. Multivariable nonlinear regression model (GAM) with backward elimination was executed to determine most important factors associated with self-rated wellness/health score. Multivariable model was adjusted for confounders. Every confounder was independently associated with both independent variable (sleep) and dependent variable (self-rated wellness/health score) and was not in the causal path between independent and dependent variable. All three conditions were to be met in order to be considered as confounding factor. The reported values of effective degree of freedom (EDF) in GAM output show the degree of curvature of the smooth in non-linear models. Value of 1 denotes a linear relationship. Values of EDF > 1 show more complex relationships between self-rated wellness/health score and sleep score. The basic residual plots were checked to assure good compliance with model assumptions. The predicted smooth functions along with the confidence intervals were plotted in multivariable GAM models. Another advantage of GAM is that it allows treating the continuous variables continuously. Data analyses were conducted using R. *p* < 0.05 was considered significant.

## Results

116 females and 26 males with Rheumatoid Arthritis were enrolled in the current study.

Demographics, lifestyle features and disease characteristics of patients are presented in Table 2. Overall, the average of BMI was in overweight range. The average night sleep duration during the week was about one hour shorter than that in weekends. Patients went to bed more than one hour later on weekends compared to weekdays (*p* < 0.0001). They also woke up more than one hour later on weekends compared to weekdays (*p* < 0.0001). Their nap duration on weekends was about half an hour longer than the nap duration on weekdays (*p* < 0.0001, Table 2). 81 patients (57%) had no snoring whereas 46 patients (32%) snored every night. Mean (SD) number of tender joints in sample was 4.42 (4.55) and mean number of swollen joints was 4 (4.26). Laboratory findings are also presented in Table 2. The most common accompanied disease was hypothyroidism (14.1%) followed by diabetes mellitus (2.8%), cardiovascular diseases (2.8%), and fatty liver (2.1%). All patients were on corticosteroids except 4% who were in remission and were not taking any medications. The maximum dose of prednisolone was 15 mg/day and the minimum dose was 1.75 mg/day. Most patients received combination therapy with prednisolone, hydroxychloroquine and methotrexate; 118 patients (83.1%) and 87 patients (61.3%) were on methotrexate and hydroxychloroquine, respectively. Other less frequent prescribed disease-modifying antirheumatic drugs (DMARD) were sulfasalazine on 40 patients (28.2%), leflunomide on 34 patients (24%), and cyclosporin A on one patient. Biologic therapy was given in the absence of response to conventional DMARD, which was predominantly adalimumab. It was added to DMARD for 14 patients (10%). Other patients’ characteristics are presented in Table 2.

**Table 2:** Demographics and Lifestyle characteristics of patients with Rheumatoid Arthritis

Patients’ characteristics	Mean	SD	Minimum	Maximum
Age, years	52.32	13	21	82
Body mass index, kg/m^2^	28.86	6.82	18.71	53.74
Time to go to bed on weekdays	22:45 PM	1	20:00 PM	2:00 AM
Time to fall asleep, Minutes	31	21	0	60
Time to go to bed on weekends	23:52 PM	1	21:00 PM	4:00 AM
Time to get out of bed on weekdays	6:08 AM	1	4:00 AM	10:00 AM
Time to get out of bed on weekends	7:20 AM	2	5:00 AM	12:00 PM
Night sleep duration on weekdays, hours	7.54	1.46	3	12
Night sleep duration on weekends, hours	8.61	1.75	4	12
Nap on weekdays, Minutes	19.75	17.87	0	60
Nap on weekends, Minutes	48.02	31.94	0	180
*How many days per week, do you have or experience the followings?*				
Difficulty falling asleep, days per week	3.24	3.25	0	7
Too early wake up﻿, days per week	5.92	2.00	0	7
Hypnotic medications use﻿, days per week	0.76	2.04	0	7
Falling asleep during the day﻿, days per week	3.65	2.81	0	7
Tired feeling upon waking﻿, days per week	3.65	3.17	0	7
Snoring﻿, days per week	2.58	3.24	0	7
Mid-sleep awakenings﻿, days per week	5.25	2.85	0	7
Headache upon waking﻿, days per week	0.98	2.13	0	7
Excessive daytime sleepiness﻿, days per week	2.06	2.94	0	7
Excessive movement during sleep﻿, days per week	1.32	2.64	0	7
Total sleep score, out of 70	29.41	12.05	3	63
Lack of energy﻿, days per week	2.75	2.98	0	7
Sadness﻿, days per week	3.32	3.21	0	7
Worry﻿, days per week	3.94	3.24	0	7
Anger﻿, days per week	3.44	3.20	0	7
Physical pain﻿, days per week	5.18	2.68	0	7
Total mood status score, out of 35	18.64	9.41	0	35
Fast food meals﻿, days per week	0.01	0.11	0	1
Red meat﻿, days per week	2.18	1.47	0	7
No fish/omega 3﻿, days per week	6.81	0.57	0	7
Less than 4/5 servings of fruits & vegetables﻿, days per week	3.96	2.05	0	7
No vitamin D tablets﻿, days per week	3.01	3.47	0	7
No magnesium tablets﻿, days per week	3.90	3.46	0	7
Total diet score, out of 42	22.35	6.44	9	42
30-minute physical activity﻿, days per week	0.30	1.35	0	7
Sociocultural activity﻿, days per week	0.27	1.26	0	7
Smoking, Pack-Year	1.63	7.77	0	40
Self-rated wellness/health score, out of 10	7.2	1.63	2	10
*Laboratory findings*				
Hemoglobin, mg/dl	12.8	2.1	9.2	30
Erythrocyte sedimentation rate, mm/h	25.5	22.5	2	100
Creatinine, mg/dl	1.2	1.7	0.2	10.7

*SD* Standard deviation

Mean (SD) self-rated wellness/health score out of 10 was 7.2 (1.63). Table 3 demonstrates the univariable linear/nonlinear association of every variable with the self-rated wellness/health score. Only the following two variables had significant univariable linear association with self-rated wellness/health score: mood status and time to fall asleep. Also, the following two variables had significant non-linear association with self-rated wellness/health score in univariable GAM analysis: sleep quality and time to go to bed on weekends. When all variables were entered in multivariable GAM regression, sleep quality, mood status, nap duration on weekdays and night sleep duration on weekends were the most significant ones. Also, age, sex, disease duration, time to go to bed on weekdays, night sleep duration on weekdays, time to fall sleep, and wake-up time on weekdays were kept in the model by backward elimination process (Table 3).

**Table 3:** Regression analyses to show univariable linear and multivariable GAM models

Predictors	Univariable association		Multivariable model	
	β (95% CI)	*P*	β (95% CI)	*P*
Age, years	− 0.008 (− 0.03–0.01)	0.50	− 0.008 (− 0.03–0.01)	0.40
Sex	0.46 (− 0.23–1.16)	0.20	0.54 (− 0.29–1.26)	0.10
Disease duration, years	0.01 (− 0.02–0.04)	0.50	0.02 (− 0.01–0.03)	0.15
Mood status score	− 0.05 (− 0.08–− 0.02)	**0.001**	− 0.04 (− 0.07–− 0.02)	**0.01**
Night sleep duration on weekdays	0.03 (− 0.15–0.21)	0.70	0.21 (− 0.04–0.50)	0.20
Night sleep duration on weekends	− 0.06 (− 0.21–0.10)	0.50	− 0.20 (− 0.41–− 0.02)	**0.03**
Time to fall asleep, Minutes	− 0.01 (− 0.03–− 0.001)	**0.03**	− 0.007 (− 0.01–0.01)	0.20
Sleep quality score	EDF* = 2.10	**0.0006**	EDF* = 2.10	**0.01**
Time to go to bed on weekdays	EDF* = 2.40	0.20	EDF* = 2.45	0.08
Wake-up time on weekdays	EDF* = 1.32	0.80	EDF* = 1.72	0.25
Nap duration on weekdays, Minutes	EDF* = 1.13	0.20	EDF* = 1.96	**0.03**
Nap duration on weekends, Minutes	− 0.003 (− 0.01–0.006)	0.50	Removed by backward elimination	
Time to go to bed on weekends	EDF = 3.51	**0.04**		
Wake-up time on weekends	0.05 (− 0.08–0.19)	0.40		
Snoring	− 0.027 (− 0.11–0.057)	0.55		
diet score	0.006 (− 0.03–0.05)	0.80		
Smoking, Pack-Years	0.01 (− 0.02–0.04)	0.60		
Physical activity	− 0.03 (− 0.24–0.18)	0.80		
Social activity	0.06(− 0.14–0.26)	0.80		
Body mass index	− 0.003 (− 0.01–0.009)	0.60		
Number of tender joints	− 0.001 (− 0.06–0.06)	0.97		
Number of swollen joints	0.01 (− 0.05–0.07)	0.70		
Extra-articular manifestations	− 0.02 (− 0.14–0.09)	0.70		
Hemoglobin level	− 0.005 (− 0.13–0.12)	0.90		
C-reactive protein	− 0.008 (− 0.03–0.01)	0.40		
Triglyceride level	− 0.001 (− 0.005–0.004)	0.80		
Fasting blood sugar	− 0.002 (− 0.11–0.006)	0.50		

The univariable outcomes presented as EDF are the products of non-linear regression models whereas β represents the linear associations. Self-rated wellness/health score was the dependent variable.* p *values < 0.05 are signified in bold

^*^EDF effective degree of freedom

**Fig. 1:**
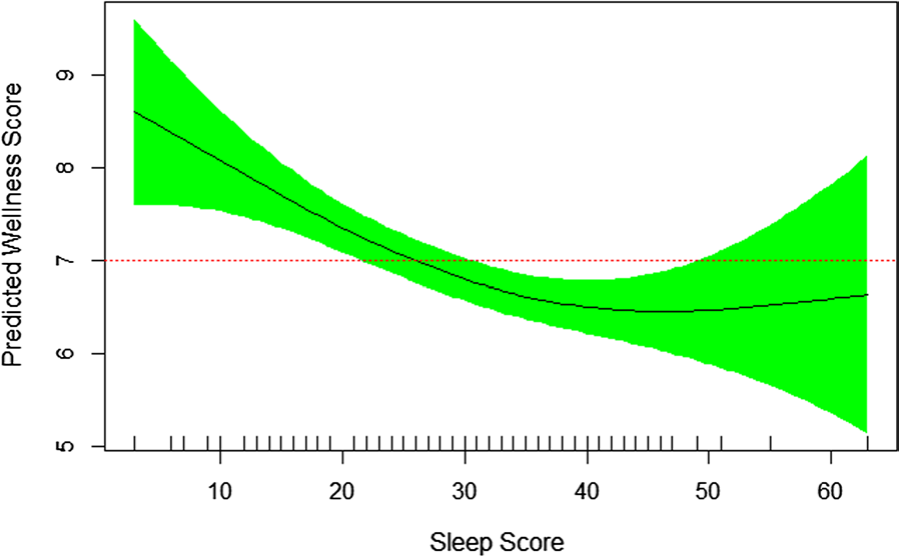
Nonlinear association of self-rated wellness/health score and sleep quality score. The higher the sleep score (the worse the sleep quality), the lower the self-rated wellness/health score (EDF = 2.10, *P* = 0.01)

**Fig. 2:**
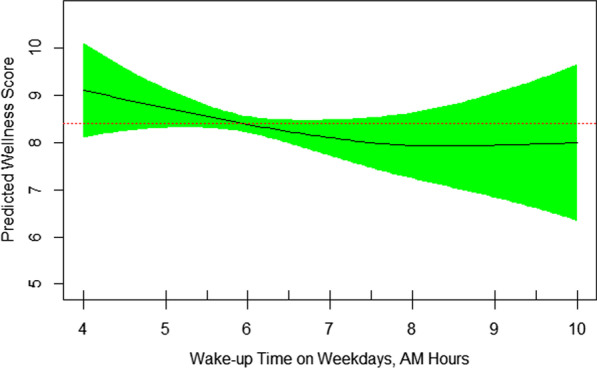
Nonlinear association of self-rated wellness/health score and wake-up time on weekdays. The later the wake-up time, the lower the self-rated wellness/health score (EDF = 1.72, *P* = 0.25)

**Fig. 3:**
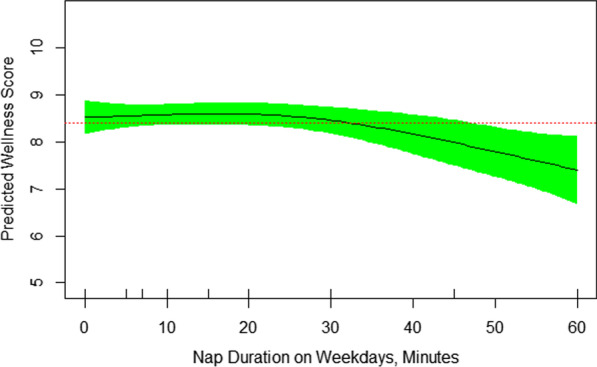
Nonlinear association of self-rated wellness/health score and nap duration on weekdays. Nap time more than 30 minutes was associated with lower self-rated wellness/health score (EDF = 1.96, *P* = 0.03)

**Fig. 4:**
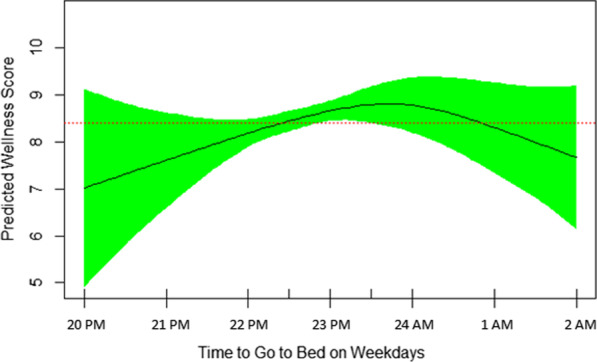
The reverse U-shape association of self-rated wellness/health score and time to go to bed on weekdays. Sleeping at 23:30 PM was associated with the highest self-rated wellness/health score (EDF = 2.45, *P* = 0.08)

Figures 1, 2, 3 and 4 were prepared based on the final multivariable GAM model presented in Table 3. They show the nonlinear association of self-rated wellness/health score with four sleep characteristics. Figure 1 demonstrates an almost 20% drop in self-rated wellness/health score when the worst sleep quality is compared with the best one. EDF of 2.1 in the final model denotes the nonlinearity of association between self-rated wellness/health score and sleep quality score (Table 3). Out of 10 measured sleep qualities, six were more prevalent in patients with rheumatoid arthritis. More than three nights per week waking up too early and mid-sleep awakening (sleep fragmentation) were reported by 86% and 76% of patients, respectively.

Other disrupted elements of sleep quality for more than three nights per week were feeling tired upon waking up, difficulty falling asleep, falling asleep during the day, and snoring, reported by 49%, 45%, 45%, and 35%, respectively. Figure 2 shows that the sooner the wakeup time, the better the self-rated wellness/health score. The nonlinearity of their relationship is also demonstrated by EDF of 1.72 in the final GAM model (Table 3). Those who woke up at 7 AM or earlier (summertime) had significantly higher mean (SD) self-rated wellness/health score than patients who woke up after 7 AM [7.23 (1.54) vs. 7.14 (2.08), respectively, *p* < 0.02). Figure 3 reveals that sleep nap longer than 30 minutes during the weekdays is associated with lower self-rated wellness/health score in patients with rheumatoid arthritis. It’s nonlinear association with self-rated wellness/health score is further revealed by EDF of 1.96 in Table 3. The mean (SD) self-rated wellness/health score in patients taking no nap vs. 5–30 minute nap vs. > 30 minute nap during the week was 7.29 (1.62), 7.39 (1.37), and 6.33 (2.05), respectively (*p* = 0.08). Figure 4 shows a reverse U-shape association between self-rated wellness/health score and time to bed during the weekdays. EDF of 2.45 in the final model shows the nonlinearity of association between self-rated wellness/health score and time to bed during the weekdays (Table 3). Patients who went to bed between 11:00 PM and midnight (summertime) had higher scores than those who went to bed either before 11:00 PM or after midnight [7.59 (1.50), 6.96 (1.61), and 6.11 (1.96), respectively, *p* = 0.008]. 55 patients went to bed between 23:00 PM and midnight, woke up at 7 AM or earlier and napped 0–30 minutes during the weekdays. Their mean (SD) self-rated wellness/health score was 7.66 (1.25) which was significantly higher than that [6.94 (1.77)] in the rest of population (N = 87) who didn’t meet one of these three criteria (*p* = 0.005). Interestingly, these 55 patients had higher number of tender joints [5.27 (5.92) vs. 3.90 (3.33), respectively], swollen joints [4.78 (5.27) vs. 3.88 (3.35), respectively], and body mass index (BMI) [34.57 (38.30) vs. 28.17 (4.95), respectively] compared to those 87 ones, although the difference was not significant in either tender joints, swollen joints or BMI. Their corresponding night sleep duration on the weekdays was 7.19 (1.05) vs. 7.76 (1.64) hours, respectively, and on the weekends, 8.65 (1.67) vs. 8.57 (1.81) hours, respectively. Other characteristics including their sleep quality scores were also similar between the two groups. When patients were divided into two groups of 7–8-h night sleep duration (N = 89) vs. those who had shorter or longer sleep duration (N = 53), no significant difference was observed between their self-rated wellness/health scores [7.22 (1.65) vs. 7.21 (1.60), respectively].

## Discussion

The current study tried to evaluate the association of self-rated wellness/health score and lifestyle dimensions in patients with rheumatoid arthritis. In building the final generalized additive model, demographic characteristics, diet, smoking, sleep, physical activity, symptoms/signs and lab data all were considered. Mood and sleep were emerged as the most important factors in the final GAM regression model associated significantly with self-rated wellness/health score in patients with rheumatoid arthritis. The model showed that too much sleep duration on weekend, more than 30 minute nap on weekdays and lower sleep quality were the main sleep indices associated with lower self-rated wellness/health score. The GAM plots demonstrated precise associations of the relationships.

The association of sleep quality and rheumatoid arthritis has been assessed by other studies [10, 11, 41–57]. Low sleep quality has been associated with increased pain, decreased physical activity and higher mental or physical fatigue [41, 43, 45, 46, 48, 49, 52]. There have been reported evidence of low sleep quality or quantity, causing symptoms of mood and increased rate of depression among patients with rheumatoid arthritis [44–46, 53]. The poor control of rheumatoid arthritis can also affect the quality of sleep in a negative manner [47, 56]. It is also worth noting that sleep deprivation can be an independent risk factor for rheumatoid arthritis and the risk of rheumatoid arthritis is increased in people with low sleep quality and short sleep duration which signifies a two-way relation between rheumatoid arthritis and sleep [42, 57].

Some aspects of sleep quality were more important in our study. Waking up too early, mid-sleep awakening (sleep fragmentation), feeling tired upon waking up, difficulty falling asleep, falling asleep during the day were the top sleep quality disruptions in patients with rheumatoid arthritis. One of the most common reported sleep disturbances experienced by patients with rheumatoid arthritis has been sleep fragmentation. Other recorded sleep disturbances were abnormalities in subjective sleep assessment, sleep latency, sleep duration, and sleep efficiency [47–49, 52–56]. A significant relationship between rheumatoid arthritis Disease Activity Score-28 or C-reactive protein and sleep quality parameters has been reported by several studies [50, 52–54, 56]. The possible mechanism of sleep and inflammatory disorders may come from an imbalance between important stages of sleep [58–61]. The mean time of going to bed on weekends in our patients was more than one hour later than the mean time of going to bed on weekdays [23:52 vs. 22:45 PM, respectively). Similarly, their mean time of wake up on weekends was more than one hour later than the mean time of wake up on weekdays [7:20 vs. 6:08 AM, respectively). This means patients in our study suffered from one-hour Delayed Sleep–Wake Phase Syndrome (weekends vs. weekdays). Whether this syndrome could explain the findings needs further studies with a proper control group. Too little deep sleep and too much REM sleep causes hormonal imbalance affecting the level of inflammation [58–61]. Whether or not the association/correlation between quality of sleep and wellbeing is different in patients with rheumatoid arthritis than in other patients [62] or a reference population without a chronic disease should be further clarified in future studies. Special clinical trials that target both sleep and stress in autoimmune disorders can also be enlightening [63].

One of the strengths of the current study was the measurement of self-rated wellness/health score as the primary outcome. Assessment of sleep quality and quantity on both weekdays and weekends was an important aspect of the current study. An additional advantage was the measurement of various lifestyle factors plus signs/symptoms/laboratory findings that were all used to build the multivariable generalized additive model. The latter explored the non-linear association between outcome and independent variables. Our study had some limitations. The cross-sectional design didn’t let launching a cause and effect association between self-rated wellness/health score and sleep quality. Longitudinal studies and inclusion of a control group may further elucidate the relationship. Self-reported frequency of snoring without monitoring by a third party could induce a bias. Diet was evaluated roughly by asking six questions that have shown associations with inflammation. A more complete diet measurement could provide deeper information about the diet index of patients.

In conclusion, mood and sleep were demonstrated as the most important factors in association with low self-rated wellness/health score in patients with rheumatoid arthritis. Several sleep characteristics showed important associations with self-rated wellness/health. Among them, sleep duration on weekend, nap duration on weekdays and sleep quality were the main sleep indices associated with self-rated wellness/health score in patients with rheumatoid arthritis in our study.

## Data Availability

All data generated or analyzed during this study are included in this article. The unidentified raw data can be provided upon request by the corresponding author, Abbas Smiley.
